# Use of low cost near-infrared spectroscopy, to predict pasting properties of high quality cassava flour

**DOI:** 10.1038/s41598-024-67299-w

**Published:** 2024-07-25

**Authors:** Mikidadi Abubakar, Peter Wasswa, Esther Masumba, Patrick Ongom, Geoffrey Mkamilo, Edward Kanju, Wilfred Abincha, Richard Edema, Karoline Sichalwe, Phinehas Tukamuhabwa, Siraj Kayondo, Ismail Rabbi, Heneriko Kulembeka

**Affiliations:** 1 Department of Agricultural Production, College of Agricultural and Environmental Sciences, Makerere University, P.O. Box 7062, Kampala, Uganda; 2 Tanzania Agricultural Research Institute (TARI), Kibaoni, Tanzania; 3 International Institute of Tropical Agriculture (IITA), Ibadan, Nigeria; 4 Kenya Agricultural and Livestock Research Organization (KALRO), Kakamega, Kenya; 5 International Institute of Tropical Agriculture (IITA), Kano, Nigeria; 6 International Institute of Tropical Agriculture (IITA), Dar es Salaam, Tanzania

**Keywords:** Pasting temperature, Peak viscosity, Breakdown viscosity, Prediction models, Early generations’ phenotyping, Calibration models, Biological techniques, Plant sciences

## Abstract

Determination of pasting properties of high quality cassava flour using rapid visco analyzer is expensive and time consuming. The use of mobile near infrared spectroscopy (SCiO™) is an alternative high throughput phenotyping technology for predicting pasting properties of high quality cassava flour traits. However, model development and validation are necessary to verify that reasonable expectations are established for the accuracy of a prediction model. In the context of an ongoing breeding effort, we investigated the use of an inexpensive, portable spectrometer that only records a portion (740–1070 nm) of the whole NIR spectrum to predict cassava pasting properties. Three machine-learning models, namely glmnet, lm, and gbm, implemented in the Caret package in R statistical program, were solely evaluated. Based on calibration statistics (R^2^, RMSE and MAE), we found that model calibrations using *glmnet* provided the best model for breakdown viscosity, peak viscosity and pasting temperature. The glmnet model using the first derivative, peak viscosity had calibration and validation accuracy of R^2^ = 0.56 and R^2^ = 0.51 respectively while breakdown had calibration and validation accuracy of R^2^ = 0.66 and R^2^ = 0.66 respectively. We also found out that stacking of pre-treatments with Moving Average, Savitzky Golay, First Derivative, Second derivative and Standard Normal variate using glmnet model resulted in calibration and validation accuracy of R^2^ = 0.65 and R^2^ = 0.64 respectively for pasting temperature. The developed calibration model predicted the pasting properties of HQCF with sufficient accuracy for screening purposes. Therefore, SCiO™ can be reliably deployed in screening early-generation breeding materials for pasting properties.

## Introduction

Cassava (*Manihot esculenta Crantz*) is an important food-security staple crop for over 800 million people in Latin America, Asia, and Africa^1^. All parts of cassava are useful, foliage are consumed as vegetables, stems as planting materials and roots for food or starch extraction due to unique starch properties. At a household level, cassava roots are consumed as fresh roots, cooked roots, baked or fried. Both fresh and dried cassava chips are used in livestock feeding. Cassava roots can be processed to make high quality cassava flour (HQCF). High quality cassava flour is used to make various products such as bread, bans, and cakes. Starch extracted from cassava can be to make biscuits. Cassava starch can be used as adhesives, can be used in paper manufacturing, in textile industry, in pharmaceutical, in cosmetic Industries and in making biodegradable products. Thus, cassava serves both as food and a raw material for industrial purposes. These diverse uses of cassava make it a potential crop contributing to food security and economic development.

In addition, compared to other important staples, cassava is a resilient crop with high tolerance to drought and poor soils. The crop widely adapt to various climates and cropping systems^2^. Fresh cassava roots contain 30–40% dry matter of which around 74–85% of the dry root weight is starch^3^. Utilizing cassava in food sectors and at the home level or applying cassava starch successfully depends on its cooking quality, notably its pasting properties^4^. The knowledge of pasting properties is an essential indicator of the cooking quality of foods and their constituents^5^. Food processors mainly apply this knowledge to optimize the ingredient levels and temperature–pressure requirements to achieve the desired product^5^.

Pasting properties are primarily evaluated using pasting curves. The pasting curves are obtained using Rapid Visco Analyser (RVA). However, the determination of pasting properties of high quality cassava flour (HQCF) with an RVA is time consuming and expensive^6^. Many breeding programs are limited with resources, so screening a large number of germplasm in early generation using RVA is a great challenge.

NIR spectra are rich in chemical and physical information about organic material and may yield valuable information about the material^7^. Using statistical and mathematical manipulation of the spectral data scientists have been able to use NIR spectroscopy to predict several traits in several crops including cassava^1,8–12^. In addition, pasting properties of rice were predicted with sufficient accuracy by NIRS based on flour spectra^6^. In prediction, calibration models are generated and are validated using reference values and spectra data. These calibration models are then used for phenotyping purposes. NIRS (SCiO™) technique had hitherto not been used for predicting pasting properties in cassava. Therefore, the aim of this study was to assess the feasibility of utilizing a portable near-infrared spectrometer (SCiO™) spectrometer in predicting pasting properties of high-quality cassava flour. SCiO™ could be a cost-effective solution in early generations’ phenotyping for pasting properties of HQCF in resource-limited cassava breeding programs.

## Materials and methods

### Genetic materials and study site

The set of genetic materials used in this study comprised of 236 cassava genotypes. Some of the genetic materials had a background from Latin America, IITA and TARI. The other genetic materials were a random collection of elite genotypes from famers’ fields in Tanzania. Genotypes were planted in single rows of ten plants each. Planting was done in two different locations namely Tanzania Agricultural Research Institute (TARI)-Ukiriguru in the lake zone and Chambezi-experimental station in the eastern zone. The genotypes were planted using Augmented design in 2020/2021 and 2021/2022 seasons. TARI-Ukiriguru is located in Misungwi District, (2° 43.1′ S, 33° 1.0′ E) at an altitude of 1198 m above sea level^13^ whereas Chambezi is located at around 6° 38′ 39′′ S, 39° 10′ 29′′ E.

### Reference data measurement

#### Sample preparation

Before reference data measurement, cassava roots were prepared into HQCF using a standard protocol developed by IITA^5^. At 12 months after planting (MAP), five fresh cassava roots that were healthy and firm were harvested from each plot at both sites. The randomly selected fresh and healthy cassava roots were then peeled and washed using tap water. The washed cassava roots for each genotype were grated into a mash separately using a mechanical grater. The mash was then placed into a clean bag and strongly squeezed to make it crumbly. The resulted product was dried in an oven at 60 °C for 24 h and stored at 4 °C waiting for further analysis.

#### Determination of HQCF pasting properties

Laboratory analysis for HQCF pasting properties determination was done at the International Institute of Tropical Agriculture (IITA), Dar es Salaam, Tanzania. The Analysis was done using Perten Rapid Visco Analyzer (RVA) Tecmaster equipment whose model number is N103802. Before RVA profiling, the stored mash was milled to obtain HQCF. Three grams (3.0 g) of HQCF of each genotype that were harvested were weighed and poured into the canister; 25 ml of distilled water was then poured into the canister’s contents. The product was thoroughly stirred to mix and then the canister was fitted into the RVA equipment. The product was heated from 50 to 59 °C with a holding time of 4 s and then cooled to 50 °C with 4 s holding time. The pasting properties of HQCF namely: peak viscosity, breakdown and pasting temperature were read from the pasting profile. These pasting properties parameters are among useful measures of pasting quality for industrial application of HQCF. Pasting temperature is associated with the energy costs during processing. Peak viscosity is an indication of water holding capacity of HQCF. Breakdown is related to paste stability^14^.

#### Spectroscopic analysis

Near infrared (NIR) spectra was collected using a highly portable molecular sensor called SCiO™. This device collects spectra on electromagnetic spectrum region between 740 and 1070 nm. SCiO sensors transmit data into a tablet through synchronization with Bluetooth. Before analysis, the device was calibrated using a built-in reference. The HQCF of each sample were placed in a container. Each sample was then scanned in triplicate (scanning the same samples at different positions). The average spectra of each sample were then used for further analysis.

### Calibration of SCiO™

#### Spectral averaging and pre-treatment

All analyses were done in R statistical software (R). The replicated spectra data were averaged using *aggregate* function of R. To remove outliers, spectra were filtered based on mahalanobis distance using *filter_spectra* function of *waves* package. Using *dplyr* package, the spectral data were matched with the reference data to ensure that both datasets had same samples.

#### Initial comparison of NIRS models developed with default tuning

In order to make informed decision about the most promising models for calibration and optimization, three machine-learning algorithms were evaluated: Lasso and Elastic-Net Regularized Generalized Linear Models (*glmnet*), Linear Model (*lm)* and Gradient Boosting Machines (*gbm*) all implemented within the *Caret* package in R statistical software. These machine learning models have been successively used elsewhere on phenomics for beans^15^. Prior to calibration, summary statistics of the reference data was computed. The reference data were combined with pre-treated spectra data using *moving-average* pre-treatment only. The sample was partitioned into calibration and validation subsets in a 7:3 ratio. This was achieved using *CreateDataPartition* function of Caret. Calibration was done using *glmne*t model implemented by Caret package in R statistical software. To ensure robust and fair comparisons, default-tuning parameters were used on the three models; all cross-validated using repeated CV of 50 folds with 20 repetitions*.* Evaluation of the models was compared based on two metrics: coefficient of determination (R^2^) and root mean square error (RMSE).

#### Optimization and comparison of glmnet and gbm calibration models

Following the results from initial model comparison, the *glmnet* and *gbm* algorithms were selected for optimization. The Spectral data for this phase underwent pre-treatment utilizing the *prospectr* package, which offers an array implemented of the following pre-treatments parameters, (1) Moving Average which removes random fluctuations around the signal (noise) that can originate from the instrument or environmental laboratory conditions, (2) Savitzky-Golay, First derivative (derivative 1) and Second derivative(derivative 2) to remove additive effects in the spectra, (3) Standard Normal Variate (SNV) which normalizes spectra to correct for light scatter, and (4) Multiplicative Scatter Correction (MSC) to remove multiplicative effects and Baseline Removal.

Two groups of calibration models were developed; (1) calibration models developed separately on each spectral pre-treatment method, and (2) models based on spectra with differently stacked pretreatments. In both these cases, *glmnet* and *gbm* models were fitted in *Caret package* in R. Both models were cross-validated using repeated CV of 50 folds with 20 repetitions. Conversely, these models have different hyper-parameter tuning factors and therefore, in *glmnet*, hyper-parameters were tuned using alpha of 0–1 and lambda of 0–1 with length of 100 while in *gbm*, hyper-parameters were tuned using number of trees equal to 500:2000, shrinkage of 01 and n.minobsinnode of 20. The best model was selected based on R^2^ and root mean square error of calibration (RMSEC) and root mean square error of validation (RMSEV). In addition, overfitting or underfitting of the model was considered in model selection.

### Compliance to IUCN policy

We, the authors of USE OF LOW COST NEAR-INFRARED SPECTROSCOPY, TO PREDICT PASTING PROPERTIES OF HIGH QUALITY CASSAVA FLOUR, hereby affirm that our research adheres to the guidelines set forth in the IUCN Policy Statement on Research Involving Species at Risk of Extinction. Additionally, we confirm compliance with the regulations outlined by the Convention on the Trade in Endangered Species of Wild Fauna and Flora. This commitment underscores our dedication to ethical research practices, conservation efforts, and the responsible management of endangered species, as outlined by these internationally recognized policies.

## Results

### Summary statistics

Summary statistics revealed a great measure of dispersion based on high values of standard deviation and range across the three traits (Table 1). Peak Viscosity had a mean value of 6052.79 Centipoise (cP), a standard deviation (SD) of 1037.19 cP, and a range of 5680.0 Cp. Breakdown viscosity had a mean of 3774.25 cP, SD of 664.57 cP and range of 4534.0 cP while pasting temperature had a mean of 75.84 °C, SD of 1.74 °C and range of 8.95 °C.

**Table 1:** Means, ranges and standard deviations (SD) of reference values for the pasting properties parameters of HQCF.

	Peak viscosity (Cp)	Breakdown (Cp)	Pasting temperature (°C)
Minimum	2320.00	1150.00	70.95
Maximum	8000.00	5684.00	79.90
Range	5680.00	4534.00	8.95
Mean	6052.79	3774.25	75.84
SD	1037.19	664.57	1.79

### Initial comparison of NIRS models developed with default tuning

**Figure 1:**
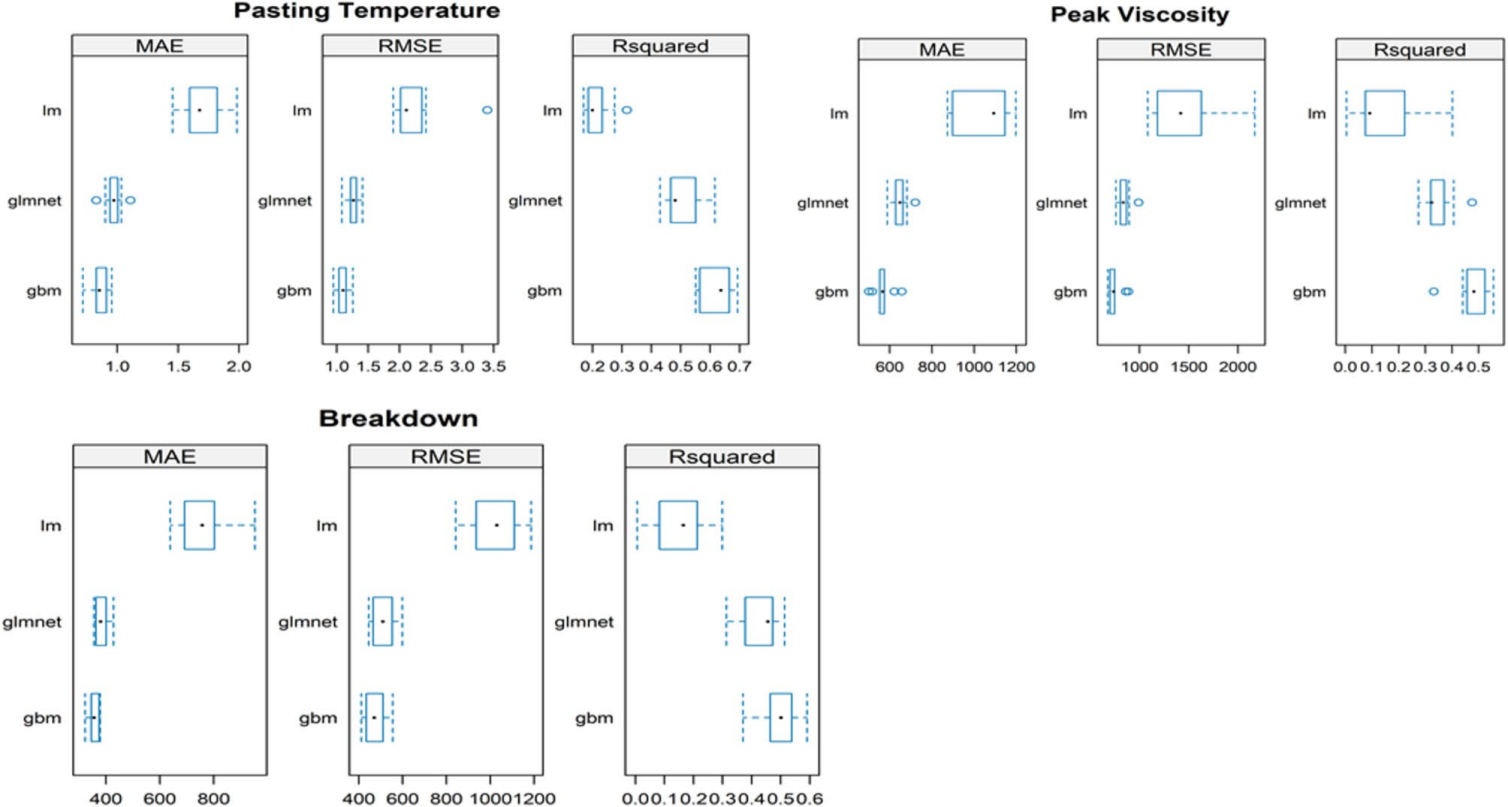
Initial model comparison statistics.

The gbm model registered the lowest MAE and RMSE; consequently, it had the highest R2 values for all three properties measured (Fig. 1). This was followed by the glmet model and, lastly, the lm model, which had higher MAE and RMSE values but the lowest R2 for all measured properties. This suggested that gbm was the most robust among the three models tested. Based on these results, two best models (gbm and glmnet) were selected for further optimization. Additionally, ANOVA results showed that the models are not statistically different (Table 2). However, model validation statistics showed that LM had the highest error values and the lowest coefficient of determination compared to glmnet and gbm (Table 3). This formed the basis for dropping LM from upstream comparison/analysis.

**Table 2:** Explanatory variable: models.

Trait	df	MS
Peak	2	1087925^ns^
Breakdown	2	78099^ns^
Pasting temp	2	1.6255^ns^

ns = not significant.

**Table 3:** Model validation statistics at initial stages of analysis.

Method	Trait	RMSE	MAE	R^2^
GLMNET	Peak	853.39	654.85	0.41
GBM	Peak	748.64	574.50	0.55
LM	Peak	1302.89	973.71	0.21
GLMNET	Breakdown	506.89	383.56	0.44
GBM	Breakdown	471.35	350.71	0.51
LM	Breakdown	1198.66	819.92	0.10
GLMNET	Pasting temp	1.26	0.97	0.51
GBM	Pasting temp	1.09	0.86	0.63
LM	Pasting temp	2.45	1.73	0.26

RMSE = Root Mean Square Error, MAE = Mean Absolute Error.

### Optimization and comparison of *glmnet* and *gbm* calibration models

More robust calibrations were achieved using *glmnet* model than *gbm* model (Tables 4 and 5). Calibrations from *gbm* models did not produce any superior model across the three studied traits. Indeed, *gbm* models were characterized with low accuracies and in any instance where calibration accuracy or validation was higher than that of glmnet, overfitting or underfitting were observed (Table 5).

**Table 4:** Calibration and validation statistics of *glmnet* model.

	R^2^ for peak viscosity		R^2^ for breakdown		R^2^ for pasting temperature	
	Calibration	Validation	Calibration	Validation	Calibration	Validation
MA	0.50 (754.11)	0.27 (919.8)	0.61 (453.17)	0.63 (398.74)	0.60 (1.19)	0.47 (1.29)
SG	0.48 (802.24)	0.39 (790.05)	0.60 (471.75)	0.52 (428.91)	0.59 (1.21)	0.52 (1.27)
D1	0.56 (718.17)	0.51 (752.31)	0.66 (424.09)	0.66 (381.76)	0.68 (1.08)	0.53 (1.20)
D2	0.53 (733.08)	0.45 (797.60)	0.66 (412.27)	0.51 (466.35)	0.65 (1.12)	0.61 (1.17)
SNV	0.26 (1053.43)	0.01 (880.28)	0.37 (578.24)	0.15 (590.88)	0.43 (1.46)	0.27 (1.67)
MSC	0.54 (1567.02)	0.23 (948.15)	0.58 (1159.91)	0.63 (417.10)	0.60 (3.20)	0.64 (1.07)
BR	0.41 (819.96)	0.29 (830.29)	0.50 (506.38)	0.47 (466.21)	0.47 (1.37)	0.45 (1.31)

MA = Moving Average, SG = Savitzky-Golay, D1 = First Derivative, D2 = Second Derivative, SNV = Standard Normal Variate, MSC = Multiplicative Scatter Correction, BR = Baseline Removal, Value in brackets is Root Mean Square Error of calibration and validation,

**Table 5:** Calibration and validation statistics of *gbm* model.

	R^2^ for Peak Viscosity (cP)			R^2^ for Breakdown (cP)		R^2^ for Pasting temp (^°^C)	
	Calibration	Validation	Calibration		Validation	Calibration	Validation
MA	0.50 (737.22)	0.53 (698.47)	0.53 (470.30)		0.72 (351.61)	0.70 (1.03)	0.51 (1.26)
SG	0.49 (774.02)	0.58 (634.71)	0.56 (460.04)		0.64 (363.17)	0.59 (1.16)	0.64 (1.06)
D1	0.40 (796.09)	0.58 (703.24)	0.55 (453.14)		0.63 (394.74)	0.59 (1.12)	0.55 (1.24)
D2	0.45 (762.35)	0.36 (892.44)	0.54(458.38)		0.57 (415.97)	0.58 (1.14)	0.52 (1.29)
SNV	0.46 (418.18)	0.61 (526.09)	0.37 (578.24)		0.15 (590.88)	0.60 (1.12)	0.52 (1.32)
MSC	0.44 (782.68)	0.39 (775.48)	0.53 (454.77)		0.44 (530.24)	0.58 (1.18)	0.58 (1.08)
BR	0.52 (718.70)	0.50 (749.95)	0.60 (423.05)		0.58 (452.01)	0.65 (1.07)	0.46 (1.34)

MA = Moving Average, SG = Savitzky-Golay, D1 = First Derivative, D2 = Second Derivative, SNV = Standard Normal Variate, MSC = Multiplicative Scatter Correction, BR = Baseline Removal, Value in brackets is Root Mean Square Error of calibration and validation, temp = temperature, cP = Centipoise.

**Figure 2:**
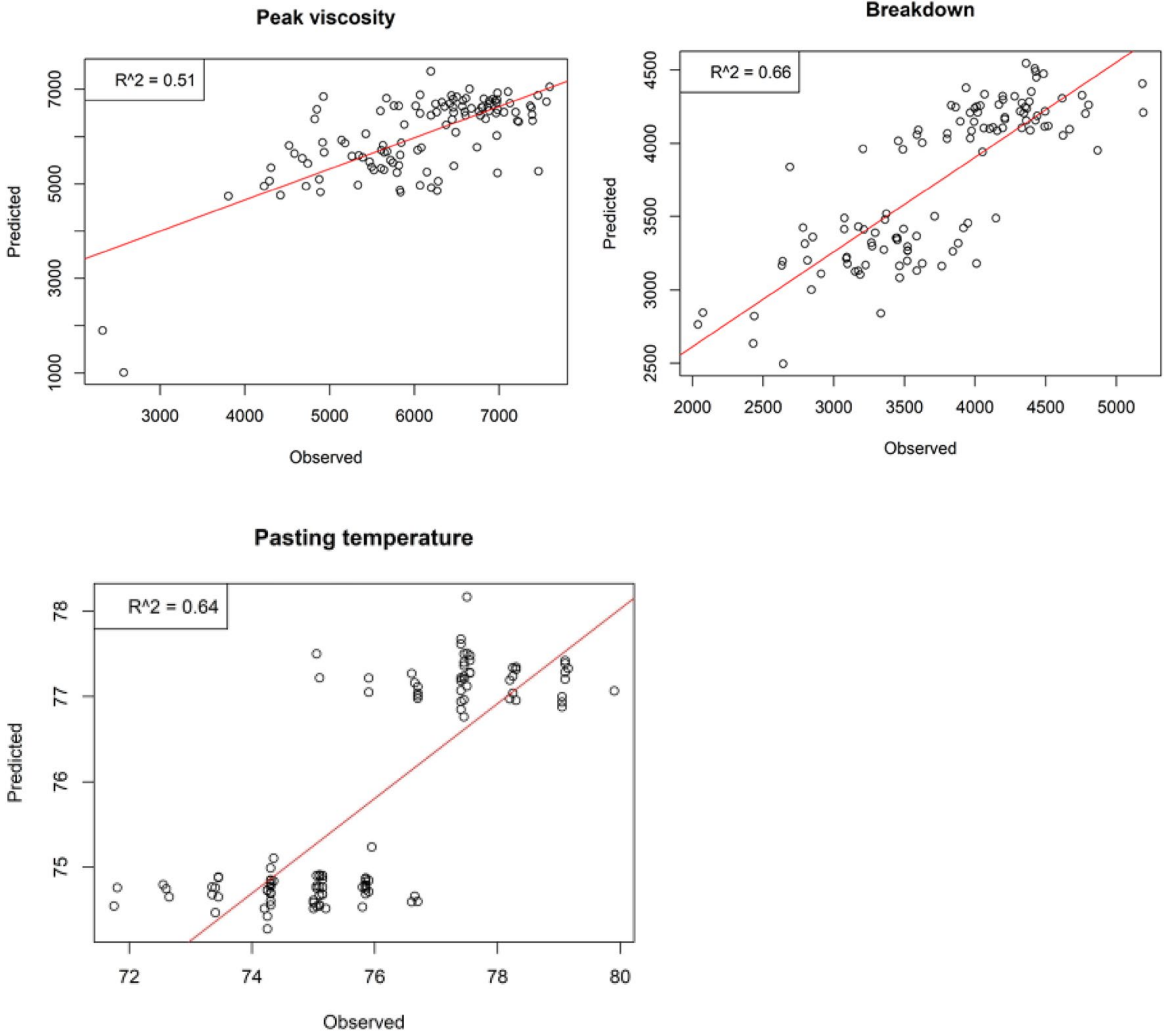
Best validation accuracy for peak viscosity and breakdown from *glmnet* model fitted using first derivative pre-treatment and for pasting temperature calibrated by stacking pre-treatment in *glmnet* model.

Robust calibration models for peak viscosity and breakdown were achieved from models developed from individual spectral pre-treatment (Table 4). Robust model for the two traits was revealed in *glmnet* model developed using *first derivative* spectral pre-treatment. Results revealed that with *glmnet* model using *fisrt derivative,* peak viscosity had calibration and validation accuracy of R^2^ = 0.56 and R^2^ = 0.51 respectively while breakdown had calibration and validation accuracy of R^2^ = 0.66 and R^2^ = 0.66 respectively (Fig. 2). Nevertheless, in all models there were some instances of low accuracies, overfitting and underfitting. Results from model calibrations using *glmnet* provided overall best model for pasting temperature (Table 6). Compared to other models, stacking of pre-treatments with Moving Average + Savitzky Golay + First Derivative + Second derivative + Standard Normal variate provided calibration model for pasting temperature with calibration and validation accuracy of R^2^ = 0.65 and R^2^ = 0.64 respectively (Fig. 2).

**Table 6:** Calibration and validation R^2^ for *glmnet* models developed by stacking of spectral pre-treatments.

	R^2^ for Peak Viscosity (cP)		R^2^ for breakdown (cP)		R^2^ for pasting temp (^°^C)	
	Calibration	Validation	Calibration	Validation	Calibration	Validation
MA + SG	0.51 (769.42)	0.38 (831.89)	0.58 (469.56)	0.56 (430.59)	0.60 (1.22)	0.50 (1.24)
MA + SG + D1	0.56 (718.89)	0.34 (790.94)	0.70 (380.23)	0.45 (519.76)	0.66 (1.10)	0.55 (1.18)
MA + SG + D1 + D2	0.48 (780.61)	0.44 (767.97)	0.63 (443.87)	0.51 (447.23)	0.66 (1.48)	0.60 (1.53)
MA + SG + D1 + D2 + SNV	0.51 (800.31)	0.38 (759.96)	0.67 (398.97)	0.40 (549.94)	0.65 (1.23)	0.64 (1.20)
MA + SG + D1 + D2 + SNV + MSC	0.52 (794.05)	0.37 (794.05)	0.65 (433.00)	0.61 (401.54)	0.68 (1.14)	0.55 (1.20)
MA + SG + D1 + D2 + SNV + MSC + BR	0.52 (755.53)	0.47 (800.77)	0.64 (421.86)	0.43 (491.68)	0.68 (1.08)	0.53 (1.23)

MA = Moving Average, SG = Savitzky-Golay, D1 = First Derivative, D2 = Second Derivative, SNV = Standard Normal Variate, MSC = Multiplicative Scatter Correction, BR = Baseline Removal, Value in brackets is Root Mean Square Error of calibration and validation, temp = temperature, cP = Centipoise.

## Discussion

Mobile NIRS has been reported to offer quick, in-field phenotyping of cassava roots for pasting properties^11^. In the context of an ongoing breeding effort, we investigated the use of a cheaper, portable spectrometer that only records a portion (740–1070 nm) of the whole NIR spectrum to predict cassava pasting properties. The findings given here show that pasting properties prediction is feasible using low-cost, portable spectrometers like the SCiO.

### Reference data

A wide range of dispersion was revealed in the reference data through the means, ranges, and standard deviations for each pasting property parameter of the calibration. Disruptive statistics for calibration and validation set was not done due to the fact that random sampling was used to create the two sets which would reorganize differently if done again. A wider range existed in each parameter measured; this shows variations between the samples which is good for calibrations purposes. Variation in near-infrared (NIR) spectroscopy reference set is of significant importance for several reasons. Calibrations variation allows for better handling of sample heterogeneity, ensuring that the models are robust and accurate across a wide range of sample compositions and properties^16^. NIR spectrometers may exhibit inherent variability due to drift, noise, or changes in environmental conditions, incorporating variation in calibrations, the models can account for instrument-specific effects, resulting in improved accuracy and reliability of predictions^17^. Sample preparation techniques, sample presentation, and ambient conditions can also influence NIR spectroscopy measurements^9^. These effects can be captured by considering variation in calibrations, leading to predictions that are more accurate. In summary, incorporating variation in NIR spectroscopy calibrations is crucial for robust and accurate predictions across a wide range of samples and measurement scenarios.

### Initial comparison of NIRS models developed using default tuning parameters

To succeed in developing robust calibration models, one has to select superior machine learning models from several available and embark on optimizing it. Herein we compared calibration accuracies of three machine-learning models namely: *glmnet, lm* and *gbm*. From this initial comparison, *gbm* appeared to be superior followed closely by *glmnet* and lastly *lm* model. However, the significance of this difference was not determined because it aimed to provide a rough picture on promising model to continue with. This result gave us the green light to drop *lm* model and optimize *glmnet* and *gbm* models.

### Comparison of glmnet and gbm models based on single spectral pretreatment

Comparison of *glmnet* and *gbm* models developed from each of the seven pre-treatment revealed that after optimization of both models through application model tuning parameters, in overall, *glmnet* provided better model for peak viscosity and breakdown than *gbm*. These results are in line with previous studies done by^18^ who reported that *glmnet* appeared to be slightly robust than *gbm* in detection of diabetes. Comparatively, *gbm* did not offer more superior model in this research. Overall best calibration model for peak viscosity had calibration and validation accuracies of R^2^ = 56 and R^2^ = 51 respectively. This model was achieved from *glmnet* calibrations based only on first derivative spectral pre-treatment. Indeed, we had other models with higher validation accuracy of R^2^ > 56. However, their calibration accuracies were very low, reflecting underfitting, which is undesirable. Looking at all calibration models for breakdown, *glmnet* model based on *first derivative* provided overall best model with calibration and validation accuracies of R^2^ = 0.66 and R^2^ = 0.66 respectively. Similarly, there were models with R^2^ > 0.66 however, they did overfit or underfit and thus undesirable. Indeed, this results comes to an agreement with reports by^19^ who published their finding based on multiple reviews that *glmnet* is more accurate compared to *gbm* models. Overall, best model for pasting temperature was identifying from *glmnet* models developed by stacking spectral pre-treatments namely; Moving Average + Savitzky Golay + First Derivative + Second Derivative + Standard Normal Variate. This pasting temperature model respectively had a calibration and validation accuracy of R^2^ = 65 and R^2^ = 64. The fact that, best calibration model for peak viscosity and breakdown viscosity were achieved using first derivative pre-treatments. This may imply that certain sole pre-treatments are able to enhance the signal of a particular trait, thus leading to robust calibration model^20^. Furthermore, the fact that pasting temperature was achieved using stacking of moving Average + Savitzky Golay + First Derivative + Second derivative + Standard Normal Variate pre-treatments could imply that the best calibration model is not always achieved through stacking more pre-treatments, since certain pre-treatment combinations reaches a specific threshold beyond which it over-corrects the spectra making it lose signal for a particular trait. This result agrees with the results which reported that raw spectra pre-processing can negatively affect the performance of near-infrared spectroscopy models prediction^10^. Generally, results reported here provide evidence that NIR could be used to predict cassava root traits. These results agree with reports by several other scientists on the feasibility of estimating many cassava traits using NIR. Demonstrated the feasibility of cassava root dry matter content prediction with low-cost, mobile spectrometers such as the **SCiO** used in our study and informed its use in routine breeding decisions^1^. Ikeogu et al.^9^ showed the feasibility of using portable Vis/NIRS device (QualitySpec Trek: S-10016) in predicting dry matter content and carotenoids in fresh cassava roots which could accelerate accurate phenotyping and general improvement of cassava. Rittiron et al.^12^ were able to show the possibility of estimating starch content in cassava roots using portable vis/NIR spectrometers operated in interactance mode in the spectral regions of 350–1050 nm for predicting the starch content of fresh cassava roots. Showed that a portable high throughput NIRS device (QualitySpec Trek: S-10016) can be used to increase breading efficiency by its quick estimation of dry matter content and total carotenoid content in fresh cassava root^8^. The calibration and prediction accuracy results that we report here indicate that the **SCiO** is a suitable alternative to expensive spectrometers for pasting properties phenotyping, especially when factoring in cost and throughput. Indeed **SCiO** is an extremely better option because it is comparatively highly portable, cheap coupled with its high throughput ability.

## Conclusions

The feasibility of utilizing **SCiO** for pasting properties estimation of high quality cassava flour was evaluated. The developed calibration model predicted pasting properties of HQCF with sufficient accuracy for screening purposes. Therefore, **SCiO** can be reliably deployed in quality screening of early generation material for pasting properties for breeding especially in resource constrained breeding programs.

## Data Availability

The data used in this study are available on GitHub at https://github.com/mikidadio/SCiO-Calibration-data.gi.
